# Analysis and Determination of Trace Metals (Nickel, Cadmium, Chromium, and Lead) in Tissues of *Pampus argenteus* and *Platycephalus indicus* in the Hara Reserve, Iran

**DOI:** 10.1155/2014/576496

**Published:** 2014-07-10

**Authors:** Sahar Mohammadnabizadeh, Alireza Pourkhabbaz, Reza Afshari

**Affiliations:** ^1^Department of Environmental Sciences, Faculty of Agriculture, University of Birjand, Birjand, Iran; ^2^Addiction Research Centre, Mashhad University of Medical Sciences, Mashhad, Iran

## Abstract

The accumulations of Cd, Ni, Pb, and Cr were measured in muscle, gill, kidney, and liver of *Platycephalus indicus* and *Pampus argenteus*. Our results indicated that all metals were found to be the highest in tissues in *P. indicus* (benthic species). Except Ni in *P. indicus*, concentrations of metals and bioaccumulation factor were in the following sequence: liver > kidney > gill > muscle. The data revealed that there is a significant negative correlation between concentrations of metals and size and age factors. The Ni and Cr levels in the muscles were higher than the maximum acceptable limit recommended by WHO and FEPA. Similarly, the concentration of Pb measured in *P. indicus* muscle exceeded the FAO standard limit.

## 1. Introduction

Heavy metal pollution of environment has become an important concern in recent years [1]. Different natural and anthropogenic sources such as industrial discharge, agricultural operations, domestic waste, burning of fossil fuels, and also geologic weathering contribute to the amount of trace metals in the environment, especially in aquatic ecosystems [2, 3]. Unlike many organic contaminants, which lose toxicity with biodegradation, heavy metals are persistent; therefore, they cannot be metabolized and are, thus, bioaccumulative. Most of them have no advantageous functions to the body and can be very toxic [4, 5]. In aquatic ecosystems heavy metals are taken up through different tissues of the fish at different levels [6, 7]. Various biotic and abiotic factors control metal bioaccumulation in fish tissues such as feeding habits, life style, fish age, gender, body mass, and physiologic conditions, as well as water temperature, pH value, and dissolved oxygen concentration [2, 8]. Metals transferred through the aquatic food webs to fish and are finally absorbed by human [9]. Numerous studies have been carried out in a number of countries to assess the presence of heavy metals in the aquatic biota, including different species of edible fish, which can often be recommended as excellent bioindicators for evaluation of the potential effect on organism health and aquatic pollution [5, 9]. Thus, knowledge of heavy metal concentrations and monitoring their bioaccumulation in fish is important with respect to bothnature management and human health.

The present study was carried out to measure the concentrations of heavy metals (nickel, cadmium, chromium, and lead) in common edible fish tissues (liver, kidney, gill, and muscle) of two species,* P. indicus* (bartail flathead) and* P. argenteus* (silver pomfret), to determine the tissues' tendency for accumulating these metals and to identify relationships between fish species and metals accumulation. Also, bioaccumulation factors of each metal and the relationships between size (weight and length) and age and metal concentrations in the tissues were determined. Furthermore, the results were evaluated according to international standards to identify any potential public health risks in order to assess the seafood consumption safety.

## 2. Materials and Methods

### 2.1. Study Area

The Hara biosphere, with an area of 85,686 hectares (26°45′ to 26°58′N; 55°30′ to 55°50′E), is located in the Southern Hormozgan province of Iran at the northeastern coast of the Persian Gulf. It is a unique water body of ecological importance due to its singular mangrove trees, great biodiversity, and its local economy. In terms of pollution, the water quality of the Hara ecosystem is influenced by various industrial outputs, discharging their wastewater directly to the water or via rivers. Release of untreated sewage from cities and various industries close to the Hara biosphere like cement plant, power plant, lead and zinc factory, paint industries, and aluminium factory and also the release of petroleum components from commercial ships, small boats, and a refinery into water make it polluted. Such contamination must be an important issue regarding the health of the aquatic ecosystem and its animals and, in turn, human's health.

### 2.2. Analytical Procedure

**Figure 1:**
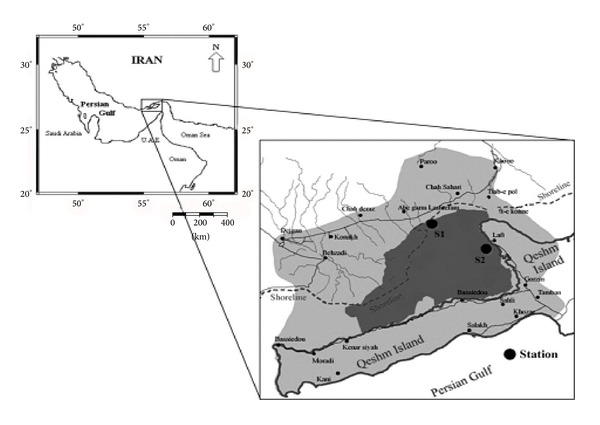
Map of the sampling location (Khamir port (s1) and Laft port (s2)) in Hara biosphere.

In this investigation, water samples and 108 fishes of two edible species, that have more consumption rate in south of Iran, were collected at two stations in the Hara biosphere water (Figure 1).

Water samples were taken from two sites in three replicates from a depth of 30 cm below the water surface and stored in 250 mL polyethylene bottles which were precleaned with detergents and soaked overnight in 10% nitric acid (Merck, Germany) and washed with double distilled water [10]. All water samples were immediately brought to the laboratory and were filtered through 0.45 *μ*m nitrocellulose membrane filters. Fish samples collections were delivered to laboratory where they were sorted by species and size. The mean and range of lengths, weights, and ages of the fish species are presented in Table 1. Each sample collected was dissected for its otolith bone (for age determination) [11], muscle, kidney, gill, and liver tissues. Lengths and weights of two fish species varied from 29.5 to 57 cm and from 180 to 618.5 g in bartail flathead and from 17.5 to 35 cm and from 100 to 680 g in silver pomfret, respectively (Table 1). About 1 g of each organ (wet weight) was weighed out in an open beaker and 8 mL of nitric acid (65%, Merck, Germany) was added. The samples were left overnight. Thereafter, 3 mL perchloric acid (70%, Merck, Germany) was added to each sample [12]. Further digestion was performed at 160°C in a sand bath on a hot plate until the solutions were clear. After cooling, mixtures were diluted to 25 mL in volumetric flasks with deionized water. Then they were filtered through 0.45 *μ*m nitrocellulose membrane filters and kept in plastic bottles at 4°C. Metal analyses were performed using a graphite furnace atomic absorption spectrometer (model AA3030 Perkin Elmer). The limits of detection were as follows: Cd (0.05 *μ*g/kg); Pb (0.8 *μ*g/kg); Ni (0.7 *μ*g/kg); and Cr (0.3 *μ*g/kg). Accepted recoveries of reference material ranged from 96% to 109%. Results were expressed as milligrams of metal per kilogram wet weight.

### 2.3. Statistical Analysis

Statistical analyses were carried out using the SPSS statistical package program. Kolmogorov-Smirnov test was accomplished to analyze the normality of data distribution. The analysis of variance (ANOVA) was used for detection of differences in metal concentrations between tissues; Tukey's honest significant difference test was employed. Furthermore, the obtained data were statistically analyzed by *t*-test for assessment of variation in metal concentrations among small and large size individuals within each species and also between two different species. Pearson's correlation coefficients were used when calculating correlations between fish length, weight, age, and concentration of metals. Moreover, bioaccumulation factor between fish and water was calculated, using the mean metal concentrations in fish and the corresponding metal concentrations for water [13].

## 3. Results and Discussion

Tables 2 and 3 show the mean and range values of the tested heavy metals in* P. indicus* and* P. argenteus* organs in different sizes of fish, along with the results of statistical comparisons of tissue metal concentrations. Also the bioaccumulation factor (BAF) of each organ is presented in these tables. The liver, kidney, gill, and muscle-metal concentrations exhibited a variation among species. Also the results show significant differences in the accumulation levels of metals in the tissues throughout the species. In general, mean concentrations in all tissues of bartail flathead and silver pomfret were as follows: Ni > Cr > Pb > Cd.

In the small size* P. indicus*, there were significant differences of Pb between all tissues (*P* < 0.05), except between the liver and kidney. In addition, significant differences were found between the liver and muscle, liver and gills, and also muscle and kidney for Cr (*P* < 0.05). The values of Cd were significantly different between all tissues (*P* < 0.05), except between the liver and kidney, and also gills and kidney. For Ni significant differences were observed between all tissues (*P* < 0.05), except between the liver and kidney, and also liver and gills. The values of Cd, Cr, Pb, and Ni in the large size bartail flathead were significantly different in all tissues (*P* < 0.01), except Ni between the liver and gills. Similarly, statistical analysis showed that the values of metals in the large size* P. argenteus* were significantly different between all tissues (*P* < 0.05), except between the kidney and gills for Ni, Cd, and Pb. In the small size silver pomfret, there were significant differences of the tested heavy metals among all tissues (*P* < 0.01), except Cd and Cr between the kidney and gills, and also muscle and gills. Moreover, there were no significant differences between the liver and kidney for Cr.

In general, in bartail flathead, the concentrations of Cd, Cr, and Pb were higher in liver with an average of 0.91, 1.49, and 1.43 mg kg^−1^, respectively, followed by kidney (Cd, 0.64; Cr, 1.1; and Pb, 1.22 mg kg^−1^), gills (Cd, 0.49; Cr, 0.79; and Pb, 0.92 mg kg^−1^), and muscle (Cd, 0.28; Cr, 0.36; and Pb, 0.63 mg kg^−1^). But the highest mean concentration of Ni was in kidney (3.18 mg kg^−1^) followed by liver (3.03 mg kg^−1^), gills (2.94 mg kg^−1^), and muscle (2.64 mg kg^−1^). In silver pomfret, the highest levels of Cd (0.61 mg kg^−1^), Pb (0.73 mg kg^−1^), Cr (1.16 mg kg^−1^), and Ni (2.26 mg kg^−1^) are found in the liver samples. Lower means were found in kidney (Cd, 0.41; Pb, 0.55; Cr, 0.89; and Ni, 1.96 mg kg^−1^), gills (Cd, 0.27; Pb, 0.42; Cr, 0.66; and Ni, 1.73 mg kg^−1^), and muscles (Cd, 0.06; Pb, 0.2; Cr, 0.3; and Ni, 1.42 mg kg^−1^), respectively. The mean concentrations of Ni (2.5–8.1), Cd (0.77–2.2), Pb (4.4–17.6), and Cr (3.8–13.8) in the fish samples from the Gulf of Aqaba in Red Sea [14] were higher than the results obtained in this investigation, while Cd levels in fish species of Saricay stream in Anatolia 0.001–0.12 [7] and middle Black Sea 0.09–0.48 [15], Cr levels for fish from Tuzla lagoon in Mediterranean region 0.26–0.82 and coastal water of Black Sea, Pb levels in fish species from the coastal water in Turkey 0.09–0.81 [16], and Ni levels 0.56–1.06 for fishes from Ataturk Dam Lake [17] were lower than our results.

While bioaccumulation factor (BAF) is different relying on the tissue, organism, and heavy metal [18], this study showed that the BAFs were higher in liver than in other tissues in both of fish species, except Ni in bartail flathead that had the highest BAF in kidney. Fernandes et al. [8] reported the highest BAFs were observed in the tissues mostly involved in metal metabolism.

Heavy metals are mostly accumulated in metabolically active organs such as liver, kidney, and gills [6]. The literature shows that the liver has a significant function in basic metabolism, contaminant storage, redistribution, detoxification, or transformation [5, 9]. Thus, liver is an excellent environmental indicator of chronic exposure to metals and water contamination [8]. Like liver, kidneys are metabolically active tissues and accumulate heavy metals of higher levels [19]. In our investigation gills had a highest concentration of heavy metals after liver and kidney. Gills are the first tissues to be exposed to suspended sediment particles, so they can be significant places of interaction with heavy metals [6, 8]. Also, the reason for high metal concentrations in the gill could be caused by the metal complexion with the mucus that is not possible to be eliminated entirely from it [20]. In contrast, results were reported from a number of fish species that the muscle tissues are not considered an active location for metal accumulation. Muscle is the organ that usually has the lowest essential and nonessential metal concentrations in fish [2, 8]. In this study Cd and Pb levels recorded in muscle samples were low, when compared to WHO and FEPA maximum recommended limits of 2.0 mg kg^−1^ in fish food [21]. But Pb levels in the bartail flathead muscles were higher than the maximum acceptable concentrations established by FAO, 0.5 mg kg^−1^ [4]. The mean concentration of Ni in the fish muscles was higher than the maximum acceptable limit of 0.5-0.6 mg kg^−1^ for food fish, recommended by WHO and FEPA [21]. Moreover, the mean concentrations of Cr exceeded these guidelines (0.15 mg kg^−1^) [21]. Based on the above findings, the consumption of fish from Hara biosphere presumably leads to Pb, Cr, and Ni inducing health hazards.

Fish age and size are important parameters when discussing metals' accumulation in fish [22]. Results show a negative correlation between all metal levels and weight, length, and age in all tissues of samples from both silver pomfret and bartail flathead (*P* < 0.01). The lower age and lower size of species accumulated more heavy metal consentrations. In various studies, negative correlations between fish size and metal concentrations were reported due to higher metabolic rates; feeding habits [23]; faster growth than metal accumulation [9]; and short residence time of these metals within the fish [1].

Distinctive species differences of metal concentrations were found in the two species (*P* < 0.01), except Cr in muscle, kidney, and gills. In this study, metal accumulations in all tissues of* P. indicus* were higher than that in* P. argenteus*. According to investigations, the heavy metal accumulation in the aquatic organism may be species dependent [2, 8]. It is a well-known fact that* P. indicus,* being a benthic fish, is continuously in contact with sediment, while the pelagic species (*P. argenteus*) showed low accumulation of the heavy metals. Aquatic animals, which have close relationship with sediment, exhibit comparatively high body concentrations of metals [19, 20]. Moreover, comparable results were reported from a number of fish species indicating that the concentration of heavy metals differed in species due to their different ecological needs [19], feeding habits [5], different metabolic activities [6], and differences in the absorptive capabilities among animals and the animals' anatomic considerations [24].

## 4. Conclusion

The following can be concluded from the present investigation.

1. The mean concentrations of heavy metals and also bioaccumulation factor were the lowest in the muscle and the highest in the liver and kidney. The results of this study reveal that liver and kidney in this species can be used as environmental indicators of metal stress. Furthermore, studies on the concentration of heavy metals in other fish tissues (gonads, intestines, skin, and heart) are recommended.
2. The accumulation of metals was more pronounced in* Platycephalus indicus* (benthic fish) than in* Pampus argenteus* (pelagic fish). Biotic factors such as behavior and feeding habits and also abiotic factors can influence the accumulation in different fish species.
3. The significant negative relationship is observed between fish age, length, weight, and accumulation of heavy metals in all tissues. Therefore, younger fishes show higher metal levels than older fishes due to higher metabolic rates.
4. Ni, Pb, and Cr levels pose a health hazard to consumers as they exceed the permissible level in muscle. Consequently, very close monitoring of heavy metal loads in Hara ecosystem is recommended in view of the possible risks to health of consumers and aquatic organisms.

## Figures and Tables

**Table 1:** Age, total length (cm), standard length (cm), and wet body weight (g) of fishes.

Factor	Bartail flathead		Silver pomfret	
	Big size (*n* = 36)	Small size (*n* = 18)	Big size (*n* = 36)	Small size (*n* = 18)
Weight	(390–618.5) 513.37 ± 86.3	(180–380) 285.92 ± 78.72	300–680 465.42 ± 60.62	(100–280) 186.67 ± 30.66
Total length	(40.5–57) 48.33 ± 6.22	(29.5–38.5) 34.25 ± 3.47	(28–35) 31.62 ± 2.7	(17.5–24) 20.83 ± 2.91
Standard length	(36–53) 44.08 ± 6.22	(25.5–34.5) 30.42 ± 3.48	(22–28) 25.25 ± 2.62	(12–18) 14.83 ± 2.69
Age	>4	<4	>4	<4

Data are represented as (range) mean ± standard deviation.

**Table 2:** Heavy metal concentrations in different organs (*µ*g/g wet wt.) of *P. indicus* and bioaccumulation factor (BAF).

Metal	Tissue	Small size		Big size		BAF
		Range	Mean ± SD	Range	Mean ± SD	
Pb	Muscle	0.68–0.78	0.73 ± 0.05^aA^	0.53–0.63	0.58 ± 0.03^bA^	4.2
	Gill	0.98–1.36	1.08 ± 0.15^aB^	0.73–0.94	0.84 ± 0.07^bB^	6.13
	Liver	1.5–2.02	1.7 ± 0.2^aC^	1.17–1.41	1.29 ± 0.08^bC^	9.53
	Kidney	1.29–1.78	1.46 ± 0.2^aC^	0.95–1.24	1.1 ± 0.08^bD^	8.13

Cd	Muscle	0.28–0.52	0.39 ± 0.11^aA^	0.13–0.33	0.22 ± 0.06^bA^	7
	Gill	0.51–0.86	0.66 ± 0.15^aB^	0.31–0.53	0.41 ± 0.07^bB^	12.25
	Liver	0.94–1.34	1.12 ± 0.17^aC^	0.64–0.91	0.81 ± 0.09^bC^	22.75
	Kidney	0.67–1.11	0.88 ± 0.19^aBC^	0.38–0.62	0.52 ± 0.07^bD^	16

Cr	Muscle	0.34–1.07	0.6 ± 0.34^aA^	0.14–0.31	0.25 ± 0.06^bA^	1.44
	Gill	0.8–1.47	1.07 ± 0.3^aAB^	0.54–0.82	0.66 ± 0.08^bB^	3.16
	Liver	1.42–3.15	2.05 ± 0.79^aC^	0.92–1.5	1.21 ± 0.17^bC^	5.96
	Kidney	1.03–2.15	1.46 ± 0.27^aBC^	0.63–1.14	0.92 ± 0.16^bD^	4.4

Ni	Muscle	2.63–3.06	2.81 ± 0.2^aA^	2.43–2.66	2.56 ± 0.07^bA^	26.4
	Gill	2.96–3.21	3.07 ± 0.11^aB^	2.79–2.93	2.88 ± 0.04^bB^	29.4
	Liver	3–3.48	3.39 ± 0.13^aC^	2.84–2.97	2.92 ± 0.04^bB^	30.3
	Kidney	3.24–3.54	3.24 ± 0.21^aBC^	2.94–3.20	3.08 ± 0.09^bC^	31.8

^a,b^Means with the same letter in row (different sizes) are not significantly different according to *t*-test.

^A,B,C,D^Means with the same letter in column (different tissues) for each metal are not significantly different according to Tukey's test.

SD: standard deviation.

**Table 3:** Heavy metal concentrations in different organs (*µ*g/g wet wt.) of *P. argenteus* and bioaccumulation factor (BAF).

Metal	Tissue	Small size		Big size		BAF
		Range	Mean ± SD	Range	Mean ± SD	
Pb	Muscle	0.24–0.4	0.34 ± 0.06^aA^	0.01–0.3	0.13 ± 0.1^bA^	1.33
	Gill	0.47–0.65	0.56 ± 0.07^aB^	0.09–0.57	0.34 ± 0.16^bB^	2.8
	Liver	0.8–1.04	0.92 ± 0.09^aC^	0.35–0.92	0.63 ± 0.18^bC^	4.87
	Kidney	0.62–0.83	0.72 ± 0.08^aD^	0.2–0.69	0.46 ± 0.16^bB^	3.67

Cd	Muscle	0.01–0.32	0.16 ± 0.1^aA^	0.001–0.03	0.01 ± 0.005^bA^	1.5
	Gill	0.2–0.54	0.37 ± 0.13^aAB^	0.01–0.56	0.21 ± 0.14^bB^	6.75
	Liver	0.55–0.95	0.79 ± 0.18^aC^	0.23–0.88	0.52 ± 0.21^bC^	15.25
	Kidney	0.36–0.74	0.54 ± 0.14^aB^	0.11–0.6	0.35 ± 0.16^bB^	10.25

Cr	Muscle	0.4–0.95	0.65 ± 0.22^aA^	0.01–0.3	0.13 ± 0.09^bA^	1.2
	Gill	0.64–1.25	0.9 ± 0.25^aAB^	0.2–0.91	0.54 ± 0.2^bB^	2.64
	Liver	1–1.86	1.41 ± 0.35^aC^	0.63–1.38	1.05 ± 0.24^bC^	4.64
	Kidney	0.88–1.48	1.13 ± 0.27^aBC^	0.44–1.2	0.77 ± 0.22^bD^	3.56

Ni	Muscle	1.44–1.8	1.62 ± 0.14^aA^	1.07–1.68	1.32 ± 0.19^bA^	14.2
	Gill	1.81–2.08	1.94 ± 0.1^aB^	2.79–2.93	1.62 ± 0.23^bB^	17.3
	Liver	2.33–2.64	2.46 ± 0.13^aC^	1.2–2	2.16 ± 0.18^bC^	22.6
	Kidney	2.07–2.31	2.19 ± 0.09^aD^	1.5–2.19	1.84 ± 0.21^bB^	19.6

^a,b^Means with the same letter in row (different sizes) are not significantly different according to *t*-test.

^A,B,C,D^Means with the same letter in column (different tissues) for each metal are not significantly different according to Tukey's test.

SD: standard deviation.
