# Corrigendum to “Analysis and Determination of Trace Metals (Nickel, Cadmium, Chromium, and Lead) in Tissues of *Pampus argenteus* and *Platycephalus indicus* in the Hara Reserve, Iran”

**DOI:** 10.1155/2020/4329071

**Published:** 2020-12-19

**Authors:** Journal of Toxicology

The article titled “Analysis and Determination of Trace Metals (Nickel, Cadmium, Chromium, and Lead) in Tissues of *Pampus argenteus* and *Platycephalus indicus* in the Hara Reserve, Iran” [1] was found to contain nearly 1000 words taken from the authors' published work [2], which was not cited. The articles measured the same elements in two different species of fish collected from the same study site. The article is as follows:

Sahar Mohammadnabizadeh, Alireza Pourkhabbaz, Reza Afshari, and Mohsen Nowrouzi, “Concentrations of Cd, Ni, Pb, and Cr in the two edible fish species *Liza klunzingeri* and *Sillago sihama* collected from Hara biosphere in Iran,” *Toxicological & Environmental Chemistry*, vol. 94, no. 6, pp. 1144–1151, 2012, doi: 10.1080/02772248.2012.693494.

The authors do not agree to the publication of this corrigendum.
